# The K-mer File Format: a standardized and compact disk representation of sets of *k*-mers

**DOI:** 10.1093/bioinformatics/btac528

**Published:** 2022-07-29

**Authors:** Yoann Dufresne, Teo Lemane, Pierre Marijon, Pierre Peterlongo, Amatur Rahman, Marek Kokot, Paul Medvedev, Sebastian Deorowicz, Rayan Chikhi

**Affiliations:** Computational Biology Department, Institut Pasteur, Université Paris Cité, F-75015 Paris, France; Univ Rennes, Inria, CNRS, IRISA—UMR, 6074 Rennes, France; Heinrich Heine University Düsseldorf Medical Faculty Institute for Medical Biometry and Bioinformatic, Düsseldorf 40225, Germany; Univ Rennes, Inria, CNRS, IRISA—UMR, 6074 Rennes, France; Department of Computer Science and Engineering, The Pennsylvania State University, State College 16802, USA; Department of Algorithmics and Software, Silesian University of Technology, Gliwice, PL-44-100 Akademicka 16, Poland; Department of Computer Science and Engineering, The Pennsylvania State University, State College 16802, USA; Department of Biochemistry and Molecular Biology, The Pennsylvania State University, State College 16801, USA; Huck Institutes of the Life Sciences, The Pennsylvania State University, State College 16802, USA; Department of Algorithmics and Software, Silesian University of Technology, Gliwice, PL-44-100 Akademicka 16, Poland; Computational Biology Department, Institut Pasteur, Université Paris Cité, F-75015 Paris, France

## Abstract

**Summary:**

Bioinformatics applications increasingly rely on *ad hoc* disk storage of *k*-mer sets, e.g. for de Bruijn graphs or alignment indexes. Here, we introduce the K-mer File Format as a general lossless framework for storing and manipulating *k*-mer sets, realizing space savings of 3–5× compared to other formats, and bringing interoperability across tools.

**Availability and implementation:**

Format specification, C++/Rust API, tools: https://github.com/Kmer-File-Format/.

**Supplementary information:**

Supplementary data are available at *Bioinformatics* online.

## 1 Introduction

Sets of *k*-mers are widely used in DNA sequence analysis, for instance in genome assembly [e.g. SPAdes (Bankevich *et al.*, 2012)], indexes of sequence aligners [e.g. minimap2 (Li, 2018)], large-scale sequence search tools (Marchet *et al.*, 2021). Often, bioinformatics tools are *k*-mer *consumers*, i.e. they take as input a *k*-mer set given by one of the *k*-mer *producers*, typically *k*-mer counters [e.g. KMC (Deorowicz *et al.*, 2013), DSK (Rizk *et al.*, 2013)]. Producers use *ad hoc* binary formats for storing *k*-mers on disk. This leads to inefficient development practices, as consumers need to write specific parsers for each producer format. Standard file formats greatly facilitate interoperability, e.g. in the case of the SAM/BAM formats (Cock *et al.*, 2015) for sequence alignment and HDF5 (Folk *et al.*, 2011) for general structured data.

We propose the K-mer File Format (KFF), an interoperable and efficient approach to store *k*-mer sets. We provide APIs in C++ and Rust, as well as file manipulation and conversion tools to facilitate inspection and integration into other tools. KFF has already been integrated in several tools: the KMC and DSK *k*-mer counters, the ESS-Compress (Rahman *et al.*, 2020) compression tool and kmtricks (Lemane *et al.*, 2022) for *k*-mer matrix construction. We present the rationale of our approach, the KFF 1.0 file format, and demonstrate the efficiency of KFF for storing *k*-mers from sequencing data.

## 2 Approach

Tools producing *k*-mer sets essentially use similar storage techniques. In Jellyfish (Marçais and Kingsford, 2011) and DSK, a *k*-mer is encoded in 2 bits per nucleotide and the entire set is stored as a succession of *k*-mers and associated data (e.g. abundances). In KMC, a more advanced format is used to reduce space and to allow fast, logarithmic time, queries (see ‘KMC file format description’ in the Supplementary Material for more details).

Recent works (Břinda *et al.*, 2021; Rahman *et al.*, 2021) demonstrated space-efficient storage of genomic *k*-mers using their *spectrum-like property* (Chikhi *et al.*, 2021), i.e. assuming that most *k*-mers originate from a set of long strings. In this *spectrum-preserving string set* representation (SPSS), what are stored are sequences longer than *k*, where each window of length *k* is a *k*-mer from the original set, and achieve a space of around 3 bits per *k*-mer [in Rahman *et al.* (2020), *k *=* *31, no counts stored]. However, the representation is non-trivial to compute and requires hours for a human genome.

We propose a space-efficient format that is fast to produce, encoding *k*-mers in binary and storing them in overlapping form. The drawback for space efficiency is that random accesses are not supported in KFF, yet they are unnecessary in the many consumer applications that only read *k*-mer sets from disk sequentially (Bankevich *et al.*, 2012; Rahman *et al.*, 2020).

## 3 Methods

**Fig. 1.**
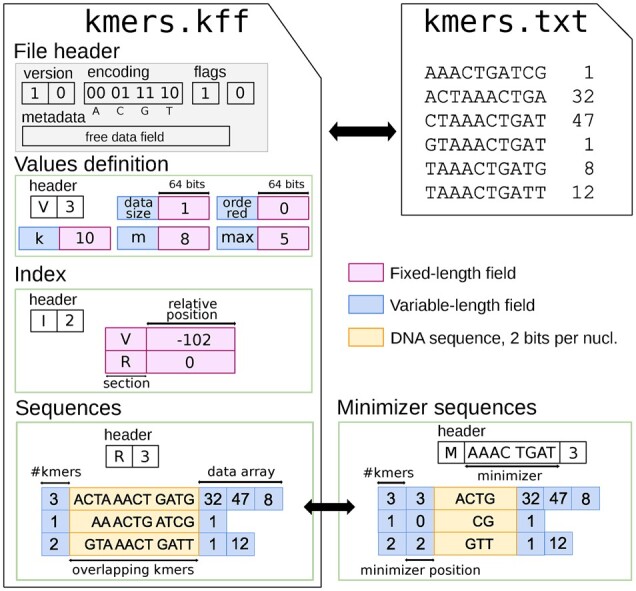
Structure of the K-mer File Format with *k *=* *10 and minimizers of size 8. Top right part: a toy *k*-mer set shown in plain text. Left part: The same *k*-mer set is represented in KFF. The top-left box is the file header and each following boxes are different sections. Bottom right part: alternatively, a Sequences section can be represented more succinctly by a Minimizer section which contains the same set of *k*-mers. For example, the first entry in the M section has sequence ACTG with its minimizer at position 3, hence it corresponds to sequence ACTAAACTGATG of size 12 (which is identical to the first entry in the R section), from which three *k*-mers can be extracted

A KFF file is composed of a short header and a succession of sections (see Fig. 1). The header contains the format version, the nucleotide 2-bit encoding (e.g. A:0, C:1, G:3, T:2), global flags to indicate whether *k*-mers are all unique and/or in canonical form, and finally a metadata section.

The rest of the file consists of sections of several types. The header of a section indicates its type. A V section defines variables that are helpful for the following sections. Actual *k*-mer sets and their associated data are stored in either sequences (R) or minimizer sequences (M) sections. In both R and M sections, longer sequences store overlapping *k*-mers, avoiding some redundancy. R sections store sequences explicitly, and the key idea of M sections is to avoid storing the minimizer sequence explicitly, and instead only indicate at which position to insert it in the stored sequence. An I section provides an index to quickly find the positions of sections within a KFF file, but its purpose is not to index *k*-mers themselves. For more details, see Supplementary Material ‘KFF file format details’ section.

The C++ and Rust APIs provide convenient ways to read and write KFF files, and in particular a high-level C++ function is provided to iterate through *k*-mers in only four lines of code.

## 4 Results

We created the kff-tools software suite on top of the C++ KFF API. It is a collection of small programs that manipulate KFF files, such as merging/splitting, validation, bucketing. They are available at github.com/Kmer-File-Format/kff-tools. These tools complement the already existing KMC tools (Kokot *et al.*, 2017) that allow more complex operations on *k*-mer sets, e.g. union, intersection and complex joins. KMC tools have further been adapted to support KFF files where *k*-mers are ordered.

To demonstrate that KFF provides significant space savings compared to other file formats, we downloaded short-read sequencing data from the chicken genome (2.8 billion distinct 32-mers) and the Human genome (5.7 billion distinct 32-mers), counted using KMC (Deorowicz *et al.*, 2013). We evaluated several file formats: naive text representation, KMC format, KFF storing *k*-mers naively, KFF where *k*-mers are compacted as super-*k*-mers (i.e. a group of overlapping *k*-mers that share the same minimizer) (see Supplementary Material ‘Experimental setup relative to kmtricks’ section) and KFF where *k*-mers are compacted using a spectrum-preserving string set (Rahman *et al.*, 2021) (see Supplementary Material ‘Experimental setup relative to ESS-Compress’ section). Full data processing details, as well as additional results using compression, are available in the Supplementary Materials.

Table 1 shows that by recording compacted super-*k*-mers with KFF, it is possible to use roughly 3× less space than with native KMC format for storing the same set of *k*-mers. In terms of running times, on the Gallus dataset using 8 threads, KMC took 9 min, KFF+sk 113 min and KFF+SPSS 900 single-threaded minutes (optimization pending). On average KFF with super-*k*-mers requires 17 bits per *k*-mer (omitting the data), while KMC uses 56 bits/*k*-mer. Using SPSS improves storage further to 5 bits per *k*-mer. Furthermore, gzip compression adds an additional 2× compression gain for KFF files and 1.25× gain for KMC files.

**Table 1.** Comparison of file sizes (in GB) for several techniques for storing 32-mers on disk: naive plain-text encoding (‘T’), KMC file format (‘KMC’), KFF file format storing one *k*-mer per block (‘KFF+naive’) or storing super-*k*-mers as created by kmtricks (‘KFF+sk’), or using *k*-mers stored as a string-preserving string set (’KFF+SPSS’)

Sample	T	KMC	KFF+naive	KFF+sk	KFF+SPSS
*Gallus gallus*	95.1	19.1	24.2	7.4	4.2
*G.gallus, gz*	19.9	15.0	16.6	4.8	2.0
*Homo sapiens*	191.0	37.7	48.5	16.8	11.1
*H.sapiens, gz*	37.9	30.6	33.8	11.9	6.4

*Note*: ‘gz’ indicates gzip compressed outputs.

In conclusion, we propose the *k*-mer set file format KFF, along with a versatile C++ and Rust API to read and write *k*-mers and a toolkit for file manipulations. We hope that KFF will boost interoperability between many software tools that use *k*-mer sets, and simultaneously improve their efficiency due to the compression features of KFF. Many suggestions and requests are emerging from discussions with the community and extensions of features to the format are currently being considered. The KFF format could for instance be used to store *k*-mer sketches, although current sketching tools store hashes on disk (Pierce *et al.*, 2019), discarding the originating *k*-mers.

## Supplementary Material

btac528_Supplementary_Data
