# Supplementary material for: The K-mer File Format: a standardized and compact disk representation of sets of k-mers
Source: Bioinformatics. 2022 Jul 29;38(18):4423–5. doi: 10.1093/bioinformatics/btac528 (PMC9477520; doi:10.1093/bioinformatics/btac528)
Supplement: btac528_Supplementary_Data [file btac528_supplementary_data.pdf]

Supplementary material

KMC file format description

In the KMC file format for storing k-mers, there are n bins of sorted k-mers. Each bin is composed of two parts: prefixes and suffixes. The first one defines for each possible prefix the position of the first and the last k-mer with this prefix in the suffix part. Thus the prefix itself does not need to be stored numerous times. The length of the prefix is calculated to optimize the space needed. In addition, for random access queries, there is an array allowing to identify in which bin the queried k-mer should be searched.

KFF file format details

All sections are composed of a *header* and a series of *blocks*. The header defines n, the number of blocks inside of the section.

In a `V` section, each block is a key/value pair: a C-style string (null-terminated) as key and a 64 bits value, e.g. ("k", 31). In a `R` section, each block contains an integer x of the number of k-mers in the block, used to determine how many bytes to read next, then $\lceil x/4 \rceil$ bytes containing a 2 bits/nucleotide sequence and finally $data\_size \times x$ bytes to store associated data in the same order as the k-mers. A `M` section contains blocks that are nearly identical to the `R` section except that an additional field indicates the position of the minimizer in the sequence. All the sequences in a `M` section share the same minimizer. These sequences are stored by deleting the minimizer in them and storing the position of where to insert it. The `I` sections are indexes of other sections. They are linked list of sections that contains blocks of pairs of values: A section type and a relative position from the end of the current section. These `I` sections allow random access over indexed sections.

The number of kmers in a block are fields encoded as big endian and the values are between 0 and a maximal value max (included) defined in the previous `V` section. This sets the size of each field as $\lceil \log_2(max)/8 \rceil$ bytes. The position in sequence fieds are encoded regarding max, m and k from the previous `V` section (m is 0 for `R` sections). Values are between 0 and $max + k - m$ and the fields are of size $\lceil \log_2(max + k - m)/8 \rceil$ bytes.

General experimental setup

In our results we applied the same protocol to both Gallus and Human samples. First we computed k-mer sets using KMC, keeping all the k-mers without any abundance cut-off. Maximal abundances are set to 255 in accordance to default KMC parameters. We executed a modified version of kmtricks specially crafted to output super-k-mers and a new version of ESS-compressor that supports KFF output to generate compacted kmer KFF files (see the next two subsections). All the outputs contains exactly the same kmers with the same counts.

Experimental setup relative to kmtricks

In kmtricks, k-mers are output in KFF format as super-k-mers (a set of overlapping k-mers that shared the same minimizer) associated with abundance vectors, in `M` sections.

The construction procedure consists of three steps:

1. Split reads into an array of super-k-mers.
2. Build a k-mer abundance table from these super-k-mers.
3. Read each super-k-mers and associate abundances to its k-mers thanks to the k-mer abundance table. If a k-mer has already been seen in another super-k-mer, the current super-k-mer is splitted into super-k-mers that contain only new k-mers.

Sources and command lines are available at `https://github.com/tlemane/kmtricks` on `kmtricks-v1.0.0-kffsk` branch.

Experimental setup relative to ESS-Compress

We extended ESS-Compress (version 3.0) to support reading from a KFF and outputting to a KFF. When the input is a KFF file, ESS-Compress runs in "UST mode," meaning that it generates a spectrum-preserving string set (SPSS) and outputs a KFF. Note that in this mode, other compression features are disabled, as they are incompatible with the KFF output format. In particular, the more advanced compression features of ESS-Compress rely on special non-nucleotide characters in the output, which KFF does not support.

When the input is a KFF file, ESS-Compress extracts the k-mers and the associated abundances from all the sections. Then, it computes the SPSS from the union of these k-mers. Next, it associates abundances to each k-mer in each sequence using Blight Marchet *et al.* (2021). It then converts this into a KFF file that has only one section, containing all the generated sequences and abundances. The ESS-compress software is available at `https://github.com/medvedevgroup/ESSCompress/`. Details on reproducing the experimental results of this paper are available at `https://github.com/medvedevgroup/ESSCompress/blob/master/kff-experiments.md`.

References

Marchet, C. *et al.* (2021). Efficient exact associative structure for sequencing data. *bioRxiv*.
